# LIPID METABOLISM CONTROLS CELL FATE DECISIONS BETWEEN CELLULAR SENESCENCE AND FERROPTOSIS

**DOI:** 10.1093/geroni/igae098.0736

**Published:** 2024-12-31

**Authors:** Christopher Wiley

**Affiliations:** Tufts University, Medford, Massachusetts, United States

## Abstract

Recent advances have identified cellular senescence as a bona fide driver of aging and several age-associated pathologies in response to various forms of stress or damage. Primary phenotypes associated with cellular senescence include a stable proliferative arrest and secretion of a mélange of biologically active molecules collectively known as the senescence-associated secretory phenotype (SASP), but senescent cells also display other key phenotypic alterations. Key among these is survival in the face of stress and damage. We recently identified multiple alterations in lipid metabolism, especially those associated with polyunsaturated fatty acids, that favor survival of senescent cells over a form of cell lytic death known as ferroptosis. Interventions that target these pathways, including inhibition of the lipid peroxidation antagonist Gpx4 or elevation of dihomo-gamma-linolenic acid (DGLA), result in lytic death of senescent cells. Elimination of senescent cells using these pathways improves phenotypic outcomes in aged mice. Finally, obese human males carrying a genetic variant with low DGLA desaturation rates are protected from accumulation of senescent cells in adipose tissue, suggesting that these interventions could be translatable to patients. Together, our results provide a new set of targets for intervening in senescence and its associated outcomes.

